# Loss of PROTEIN TARGETING TO STARCH 2 has variable effects on starch synthesis across organs and species

**DOI:** 10.1093/jxb/erac268

**Published:** 2022-06-18

**Authors:** Alexander Watson-Lazowski, Emma Raven, Doreen Feike, Lionel Hill, J Elaine Barclay, Alison M Smith, David Seung

**Affiliations:** John Innes Centre, Norwich Research Park, Norwich, UK; John Innes Centre, Norwich Research Park, Norwich, UK; John Innes Centre, Norwich Research Park, Norwich, UK; John Innes Centre, Norwich Research Park, Norwich, UK; John Innes Centre, Norwich Research Park, Norwich, UK; John Innes Centre, Norwich Research Park, Norwich, UK; John Innes Centre, Norwich Research Park, Norwich, UK; University of California, Davis, USA

**Keywords:** Barley, *Brachypodium*, carbohydrate metabolism, PTST2, starch, starch granule initiation, wheat

## Abstract

Recent work has identified several proteins involved in starch granule initiation, the first step of starch synthesis. However, the degree of conservation in the granule initiation process remains poorly understood, especially among grass species differing in patterns of carbohydrate turnover in leaves, and granule morphology in the endosperm. We therefore compared mutant phenotypes of *Hordeum vulgare* (barley), *Triticum turgidum* (durum wheat), and *Brachypodium distachyon* defective in PROTEIN TARGETING TO STARCH 2 (PTST2), a key granule initiation protein. We report striking differences across species and organs. Loss of PTST2 from leaves resulted in fewer, larger starch granules per chloroplast and normal starch content in wheat, fewer granules per chloroplast and lower starch content in barley, and almost complete loss of starch in *Brachypodium*. The loss of starch in *Brachypodium* leaves was accompanied by high levels of ADP-glucose and detrimental effects on growth and physiology. Additionally, we found that loss of PTST2 increased granule initiation in *Brachypodium* amyloplasts, resulting in abnormal compound granule formation throughout the seed. These findings suggest that the importance of PTST2 varies greatly with the genetic and developmental background and inform the extent to which the gene can be targeted to improve starch in crops.

## Teaser

Loss of PTST2, a protein involved in starch granule initiation, had highly variable effects between three grass species on carbohydrate metabolism and physiology of leaves and granule morphology of seeds.

## Introduction

Starch is a major storage carbohydrate in leaves and non-photosynthetic organs of plants. Transitory starch accumulates in leaf chloroplasts during the day and is degraded at night to provide substrates for growth and maintenance (Smith and Zeeman, 2020). Storage starch accumulates in amyloplasts of seeds and storage organs, and is used to fuel germination or regrowth (Lloyd and Kossmann, 2015). Starch is synthesized as semi-crystalline, insoluble granules composed of two glucose polymers: amylopectin and amylose. The biosynthesis of these polymers is relatively well understood and highly conserved between different species and organs (Smith and Zeeman, 2020). However, the process by which starch granules are initiated is poorly understood. Variation in this process could underlie the vast diversity in the number and morphology of granules between different species and organs (Seung and Smith, 2019; Chen *et al.*, 2021). For example, between organs, the transient starch granules in leaves differ in size, morphology, and abundance from storage starch granules. Between species, a remarkable diversity in starch granule morphology is evident in the endosperm of grass seeds, in which the starch granules of different species can be categorized as: (i) ‘simple’—often with a unimodal size distribution and arising from a single granule initiation within each amyloplast; (ii) ‘compound’—clusters arising from multiple granule initiations within each amyloplast; and (iii) ‘bimodal’—where a single, large A-type granule with distinctive flattened morphology initiates in each amyloplast early in seed development and smaller, spherical B-type granules initiate later in amyloplast stromules (Parker, 1985; Langeveld *et al.*, 2000; Matsushima *et al.*, 2013).

Recent work, largely in Arabidopsis, has identified proteins required for normal granule initiation, including PROTEIN TARGETING TO STARCH 2 (PTST2) (Seung and Smith, 2019; Mérida and Fettke, 2021). PTST2 is a highly conserved protein in plants that contains coiled-coils and a carbohydrate-binding module 48 (CBM48) domain at the C-terminus. In Arabidopsis leaves, loss of PTST2 does not affect total starch content, but greatly reduces the number of starch granules per chloroplast (Seung *et al.*, 2017). Most chloroplasts in Arabidopsis *ptst2* mutants contain a single, large granule in place of multiple smaller granules, suggesting that PTST2 promotes granule initiation. It may do so by delivering maltooligosaccharides as substrates for another granule initiation protein, STARCH SYNTHASE 4 (SS4) (Seung *et al.*, 2017). Consistent with this model, several mutants in Arabidopsis with altered maltooligosaccharide levels have altered starch granule numbers (Malinova *et al.*, 2014; Mérida and Fettke, 2021). Orthologues of PTST2 in rice (*Oryza sativa*) and barley (*Hordeum vulgare*) are known as FLOURY ENDOSPERM 6 (*Os*FLO6 and *Hv*FLO6), while orthologues in durum (*Triticum turgidum*) and bread wheat (*Triticum aestivum*) are named B-GRANULE CONTENT 1 (*Tt*BGC1/*Ta*BGC1). Leaves of rice mutants deficient in *Os*FLO6 also have reduced numbers of enlarged starch granules per chloroplast (Zhang *et al.*, 2022). In cereal endosperm, PTST2 orthologues are required for correct compound and bimodal initiation patterns. Loss of the PTST2 orthologue in rice (in the *Osflo6* mutant) results in heterogenous amyloplasts with highly variable numbers of initiations within each compound granule (Peng *et al.*, 2014). Its loss in barley and wheat (in the *Hvflo6*, *Ttbgc1*, and *Tabgc1* mutants) results in A-type granules with abnormal morphology and failure to produce B granules (Suh *et al.*, 2004; Chia *et al.*, 2020). The impact of loss of PTST2 on endosperm starches with uniform, simple granules is not known.

The ability of PTST2 to alter granule morphology in these various species makes it a promising target gene for applications in modifying seed starch (Chen *et al.*, 2021). However, this requires a greater understanding of the apparently disparate functions of PTST2 in different species and organs, and more knowledge of how it affects transient starch in leaves and vegetative growth. To shed more light in these areas, we isolated a *ptst2* mutant of *Brachypodium distachyon* and compared its leaf and seed phenotypes with those of mutants of wheat and barley lacking PTST2 orthologues. Although all three species are in the family Pooideae, *Brachypodium* differs from wheat and barley in both its diel carbohydrate metabolism in leaves and its granule initiation pattern in seeds. Although leaves of all three species turn over both starch and sucrose over the day–night cycle, *Brachypodium* turns over approximately equal amounts of starch and sucrose (Müller *et al.*, 2018, Preprint), whilst wheat and barley leaves typically turn over three times less starch than sucrose (Gordon *et al.*, 1980; Shaikh *et al.*, 2000; Müller *et al.*, 2018, Preprint). *Brachypodium* also produces simple starch granules in the endosperm, rather than bimodal starch granules as in wheat and barley (Chen *et al.*, 2014). Our results demonstrate that the loss of PTST2 reduces the number of granules initiated in leaves of all three species, but this has differing effects on growth and metabolism between species. There was a dramatic decrease in growth and photosynthetic rates in the *Brachypodium* mutant, but the phenotype in wheat and barley mutants was much less severe. In contrast, loss of PTST2 causes supernumerary starch granule initiations in the *Brachypodium* endosperm, leading to compound starch granules, and suggesting that PTST2 is also required for the correct formation of simple starch granules. Loss of PTST2 in *Brachypodium* was also associated with large transcriptional differences between mutant and wild-type seeds during development.

## Materials and methods

### Plant material

The *Brachypodium distachyon ptst2* mutant line (JJ20993) was sourced from the Joint Genome Institute *Brachypodium* T-DNA collection (Bragg *et al.*, 2012). The material received was segregating for a T-DNA insertion allele in exon 1 of the PROTEIN TARGETING TO STARCH2 gene (*PTST2*; Bradi1g12200), 247 nucleotide bases downstream of the start site (Supplementary Fig. S1A) within the Bd21-3 background. The exact insertion site was confirmed via PCR and Sanger sequencing (Supplementary Fig. S1B). Plants homozygous for the insertion allele were identified by PCR-based genotyping using the primers in Supplementary Fig. S1C. Bd21-3 was used as the *B. distachyon* wild-type control. The *Hordeum vulgare* (barley) mutant was Franubet (*Hvflo6*), which contains a premature stop codon mutation in PTST2 (Suh *et al.*, 2004; Saito *et al.*, 2018). The wild-type control used was the unmutagenized Nubet background. The *Triticum turgidum* (tetraploid durum wheat) mutant was *bgc1-3* (*Ttbgc1*; in cultivar Kronos) (Hawkins *et al.*, 2021). This line has a premature stop codon mutation in the A genome copy and a splice site mutation in the B genome copy. The wild-type segregant of the *bgc1-3* line was used as the wild type for wheat.

### Genome sequencing and insert identification

Genome sequencing was used to confirm that the *Bdptst2* mutant contained a single insertion at the *BdPTST2* locus (Supplementary Fig. S1D). Genomic DNA extracted from leaves was fragmented to an average size of ~350 bp, and a DNA library was created using established Illumina paired-end protocols. The Illumina Novaseq 6000 platform (Illumina) was used for genomic DNA sequencing at Novogene (Cambridge, UK) to generate 150 bp paired-end reads with a minimum coverage of 4× for ~98% of the genome (mean coverage of 30×). Sequencing reads were aligned to the *B. distachyon* v3.1 reference genome (Vogel *et al.*, 2010) using the Burrows–Wheeler Aligner (BWA) with default parameters (Li and Durbin, 2009). Subsequent processing including duplicate removal was performed using SAMtools (Li *et al.*, 2009) and PICARD (http://picard.sourceforge.net). T-DNA insert locations were identified using VirusFinder (Wang *et al.*, 2013).

### Growth conditions and biomass quantification

Plants were grown in controlled-environment chambers at 60% relative humidity and a light intensity of 150 μmol photons m^−2^ s^−1^. For leaf analyses, plants were grown in 16 h light at 24 °C and 8 h dark at 18 °C. For seed analyses, plants were grown in 20 h light at 24 °C and 4 h dark at 18 °C, which yielded a higher number of seeds per plant than 16 h light. Biomass measurements were taken at 35 d after germination.

### Gas exchange

Gas exchange measurements were taken using a 6400XT portable photosynthesis system (Li-COR) 26–30 d after germination on young fully expanded leaves. Measurements of the CO_2_ assimilation rate under growth conditions were made under the same conditions as for the growth chamber: CO_2_ concentration 400 ppm, light intensity 150 µmol m^−2^ s^−1^, leaf temperature 24 °C. Measurements of the CO_2_ assimilation rate under high light intensities (1500, 1800, and 2000 μmol m^−2^ s^−1^; CO_2_ concentration 400 ppm; leaf temperature 24 °C) were made to determine if 1500 μmol m^−2^ s^−1^ represented saturating light for each species under our growth conditions. The responses of the CO_2_ assimilation rate to step increases in light intensity (AQ) were measured under constant conditions of 400 ppm CO_2_, leaf temperature 24 °C. AQ measurements were taken after an acclimation time of 60–120 s at increasing light intensities (0, 30, 50, 75, 100, 150, 200, 500, 700, 1000, 1200, and 1500 μmol m^−2^ s^−1^).

The response of the CO_2_ assimilation rate to step increases of intracellular CO_2_ (*A*/*C*i) was measured at a saturating light intensity of 1500 µmol m^−2^ s^−1^, and leaf temperature 24 °C. The *A*/*C*i curves were carried out using the exact CO_2_ step increases and decreases outlined in Ainsworth *et al.* (2002). Maximum rates of carboxylation (*V*_cmax_) and electron transport (*J*_max_) were calculated from *A*/*C*i curves using the ‘Plantecophys’ package in R by fitting the raw data to a Farquhar, von Caemmerer, and Berry photosynthesis model (Farquhar *et al.*, 1980; Duursma, 2015). Before each measurement, the leaf was allowed to stabilize for 10–15 min until it reached a steady state of CO_2_ uptake. All measurements were taken 5–11 h after the end of the night.

### Leaf starch/sugar extraction and quantification

The method used for leaf starch quantification was previously described in Seung *et al.* (2017). Briefly, 2 cm sections from the middle of a young fully expanded leaf at 30 d after germination were collected and weighed. Samples were homogenized in 0.7 M perchloric acid and centrifuged at 20 000 *g*. For starch measurement, the pellet was washed three times in 80% (v/v) ethanol and resuspended in water. The starch in the pellet was gelatinized at 95 °C for 15 min and digested to glucose at 37 °C using α-amylase and amyloglucosidase (Roche) for 2 h. Starch content (in glucose equivalents) was determined by glucose assay with a hexokinase/glucose-6-phosphate dehydrogenase-based spectrophotometric method (Roche). For sugar measurements the supernatant was neutralized to pH 6–7 using 2 M KOH, 400 mM MES. Glucose was assayed as above, then fructose and sucrose were assayed by successive additions of phosphoglucose isomerase (Roche) and invertase (Sigma).

### Chlorophyll quantification

Chlorophyll content was quantified 21 d after germination. Leaf segments (2 cm) from the middle of a young fully expanded leaf were harvested. Samples were weighed, and then placed into 80% acetone at 4 °C for 3 d. Chlorophyll content was quantified by measuring the UV-vis of the acetone solution at wavelengths of 645 nm and 663 nm using a spectrophotometer (DS-11 FS plus, Novix). Total chlorophyll content was calculated based on sample absorbance at 645 nm and 663 nm as outlined in Arnon (1949).

### Seed starch purification and quantification

Starch granules were purified from mature seeds that were soaked overnight in 0.5 ml of ddH_2_O at 4°C, then homogenized in a ball mill at 30 Hz for 1 min. The homogenates were filtered through a 20 µm nylon mesh then centrifuged at 3000 *g* for 5 min. The pellet was resuspended in ddH_2_O and centrifuged at 2500 *g* for 5 min over a cushion of 90% (v/v) Percoll, 50 mM Tris–HCl, pH 8. The pellet was washed twice in 50 mM Tris–HCl, pH 6.8, 10 mM EDTA, 4% SDS (v/v), 10 mM DTT, while removing any remaining debris using selective resuspension. Finally, the pellet was washed twice with ddH_2_O.

For seed starch quantification, grains were ground to a powder using a ball mill at 30 Hz for 1 min. The powder (5–10 mg) was suspended in 80% ethanol, and starch was hydrolysed to glucose using reagents from the Total Starch Assay kit (Megazyme, K-TSTA). Briefly, thermostable α-amylase in 100 mM sodium acetate, pH 5, was added and incubated at 99 °C for 7 min. Amyloglucosidase (Roche) was added before further incubation at 50 °C for 35 min. The sample was then centrifuged at 20 000 *g* for 10 min to separate insoluble material. Glucose was measured in the supernatant as described above and starch content calculated in glucose equivalents.

### Starch granule size distribution

For analysis of granule size distributions, the starch was suspended in Isoton II (Beckman Coulter), and relative volume versus diameter plots were generated using a Multisizer 4e Coulter counter (Beckman Coulter) with a 30 µm aperture tube. A minimum of 50 000 granules were measured per sample.

### ADP-glucose quantification

The youngest fully expanded leaves (*n*=4) of 30-day-old plants were harvested 15–30 min prior to the end of the day. Care was taken not to shade leaves prior to snap-freezing using liquid nitrogen. Metabolites were extracted using chloroform/methanol as previously described (Arrivault *et al.*, 2009). ADP-glucose was quantified in the extracts using a Vanquish UHPLC equipped with a QExactive orbitrap mass spectrometer (Thermo). Separation was by Hilic chromatography on a 100 × 2.1 mm 2.6 μm Accucore amide column (Thermo Fisher) using the following gradient of solvent A (water containing 26.5 mM formic acid, 7.2 mM ammonia) versus solvent B (90% acetonitrile with the same buffer concentrations) run at 0.5 ml min^−1^ and 40 °C: 0 min, 86% B; 10 min, 62% B; 12 min, 62% B; 12.1 min 86% B; 17.1 min, 86% B. Positive electrospray MS spectra were collected from *m/z* 200 to 2000 at a resolution of 70 000, from which extracted ion chromatograms (tolerance 5 ppm) of the hydrogen adduct ([M+H]^(+)^ 590.0895) were taken for external standard quantification. Identity was confirmed using data-dependent MS2 with an isolation width of *m/z* 4.0 and 30 + 50% collision energies (combined in one scan). The instrument was calibrated according to the manufacturer’s instructions, and spray chamber conditions were 3000 V spray voltage, 350 °C capillary temperature, 35 units of sheath gas, 10 units of aux gas.

### Microscopy

For observation of granule morphology using SEM, mature seed sections were first fixed in 2.5% (v/v) glutaraldehyde in 0.05 M sodium cacodylate, pH 7.3 at 4 °C. Samples were then dehydrated in ­ethanol ­before critical point drying. Once dried, samples were mounted onto stubs and sputter coated with gold prior to imaging in a Nova NanoSEM 450 (FEI). For sections of leaves/developing grains: leaf segments were excised from approximately halfway along the length of the youngest fully expanded leaf at 30 d after germination and fixed in 2.5% (v/v) glutaraldehyde in 0.05 M sodium cacodylate, pH 7.3 at 4 °C. Developing grains [3 and 6 days post-anthesis (DPA)] were cut in half before immersion in the same fixative solution. Using an EM TP embedding machine (Leica, Milton Keynes, UK), samples were post-fixed in 1% (w/v) OsO_4_ in 0.05 M sodium cacodylate for 1 h at room temperature, dehydrated in ethanol, and infiltrated with LR White resin (London Resin Company). LR White blocks were polymerized at 60 °C for 16 h. For light microscopy, semi-thin sections (~0.5 µm) from the blocks were stained with periodic acid–Schiff’s reagent as described in Hawkins *et al.* (2021) and/or 0.5% (w/v) toluidine blue for 30 s, prior to mounting in Histomount for visualization. Sections were imaged with a DM6000 (Leica) or AxioObserver Z1 (Zeiss) microscope. For TEM, ultrathin sections (~90 nm) were placed on formvar- and carbon-coated copper grids (EM Resolutions, Sheffield, UK). The sections were stained with 2% (w/v) uranyl acetate for 1 h and 0.5% (w/v) lead citrate for 1 min, washed in distilled water, and air dried. Sections were imaged on a Talos 200C TEM (FEI) at 200 kV and imaged using a OneView 4K×4K camera (Gatan). Starch granule number and cross-sectional area were calculated using ImageJ (Schindelin *et al.*, 2012).

### RNA sequencing and bioinformatics

Spikelets were harvested at 6 DPA from plants at the middle of the light period and snap-frozen in liquid nitrogen. Seeds were extracted on dry ice. The lemma was not removed to limit damage to the seed and avoid excessive thawing. RNA was extracted using the RNeasy PowerPlant kit (Qiagen), incorporating an on-column DNase digest. Following quality control, library preparation with poly(A) selection was carried out at Novogene (Cambridge, UK). RNA sequencing was then carried out using a NovaSeq 6000 machine (Illumina), returning ~30 million 150 bp paired-end reads per sample.

Raw reads were first filtered to remove reads with adapter contamination or low quality. Reads were then aligned to the *B. distachyon* v3.1 reference transcriptome (Vogel *et al.*, 2010) using HISAT2 (Kim *et al.*, 2019). HTSeq (Anders *et al.*, 2015) was used to generate normalized counts [fragments per kilobase of transcript per million mapped reads (FPKM)] using union mode. Significantly differentially expressed transcripts were identified using DESEQ2 (Love *et al.*, 2014), with significance cut-offs of false discovery rate (FDR) ≤0.05 and log_2_ fold change (FC) >1. To assess functional enrichment of the differentially expressed transcripts, over-representations of Gene Ontology (GO) categories were assigned using AgriGo v.2.0 (Tian *et al.*, 2017). Singular enrichment analysis was implemented using the *B. distachyon* v3.1 reference for GO representation, a Fisher’s statistical test, a Yekutieli adjustment for multiple testing, and an adjusted *P*-value cut-off of ≤0.05. Disposable (as in also represented by a child GO term) GO categories were identified using REVIGO and discarded to remove redundancy (Supek *et al.*, 2011).

### Statistics

Welch’s *t*-tests were used when a single comparison between two groups was made. Two-way ANOVA tests were used in R (R CoreTeam, 2013) when multiple groups were compared (with species and genotype as factors). Each variable was first tested for normality using the Shapiro–Wilks test before analysis, then, if necessary, transformed using the log or squared function in R. For the two-way ANOVA, if a significance value of ≤0.05 was returned, a Tukey post-hoc test was used to identify which groups were significantly different.

## Results

### Diverse effects of *ptst2* mutations on leaf metabolism

To compare the role of PTST2 in leaves of different grass species, we obtained mutants defective in PTST2 orthologues of barley (*Hordeum vulgare*; *Hv*), durum wheat (*Triticum turgidum*; *Tt*), and *Brachypodium distachyon* (*Bd*). The barley (*Hvflo6*) and wheat mutants (*Ttbgc1*) are those described in Saito *et al.* (2018) and Hawkins *et al.* (2021), respectively, whereas the *Brachypodium Bdptst2* mutant carrying an insertion in exon 1 was isolated from a T-DNA mutant collection (Supplementary Fig. S1A–C). Whole-genome sequencing showed that this was the only T-DNA insertion in the mutant (Supplementary Fig. S1D).

**Fig. 1.**
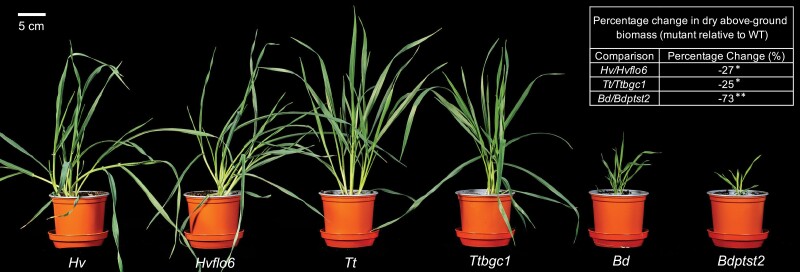
Growth phenotypes of *ptst2* mutants from three species. Photograph of wild-type (WT) and *ptst2* plants of *H. vulgare* (*Hv* and *Hvflo6*), *T. turgidum* (*Tt* and *Ttbgc1*), and *B. distachyon* (*Bd* and *Bdptst2*) at 28 d after germination. Scale bar=5 cm. Percentage change in biomass is calculated from measurements of dried above-ground biomass (*n*=4) at 28 d after germination and is shown as mutant relative to wild type. An asterisk represents a significant change (*P≤0.05 and **P≤0.001) calculated using a Welch’s *t*-test.

**Fig. 2.**
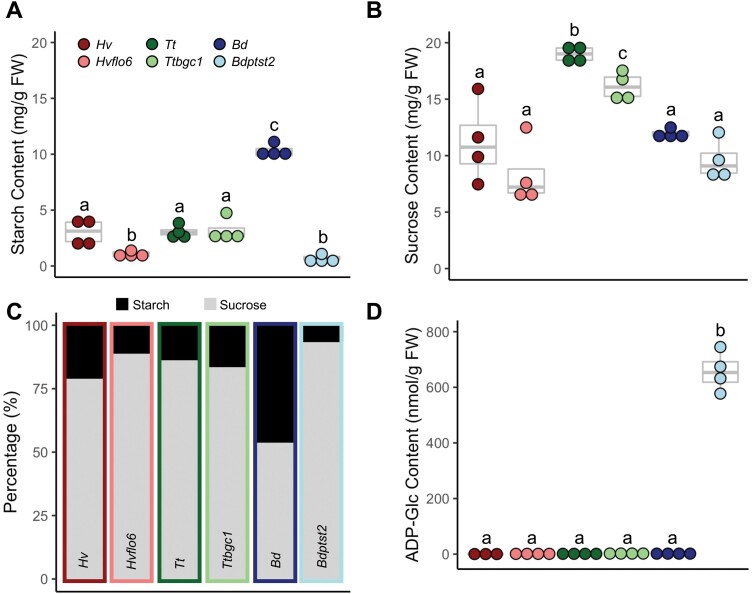
Carbohydrate quantification in leaves of *ptst2* mutants. (A) Starch and (B) sucrose content of leaves harvested at the end of the day for both the wild type and *ptst2* mutants of each species (*n*=4). (C) The percentage of starch and sucrose within the leaves, calculated from the values from (A) and (B). (D) ADP-glucose content of leaves harvested at the end of the day from each species for both the wild type and *ptst2* mutants (*n*=4). Barley [*H. vulgare* (*Hv*); *Hvflo6*], wheat [*T. turgidum* (*Tt*); *Ttbgc1*], and *Brachypodium* [*B. distachyon* (*Bd*); *Bdptst2*]. Each dot represents a biological replicate, each harvested from a different plant. Measurements for starch and sucrose were taken from the same leaf sample. Box and whisker plots show the median, upper quartile, and lower quartile. Significance is indicated where two letters differ (*P*≤0.05) calculated using a two-way ANOVA and a Tukey’s post-hoc test.

Under our growth conditions, all mutant lines had significantly reduced growth (*P*≤0.05; Fig. 1). However, in comparison with wheat and barley lines (25% and 27% reductions in dry above-ground biomass, respectively), the *Bdptst2* mutant had severely reduced growth (a 73% reduction in dry above-ground biomass) and had pale leaves (Fig. 1). Visual screening of 32 progeny from a heterozygous plant showed that this pale-leaf phenotype was recessive and cleanly segregated with the *Bdptst2* T-DNA allele. We measured leaf starch and sucrose contents at the end of day in the leaves of each species (Fig. 2A, B). As expected from previous reports, *Brachypodium* had a much higher ratio of starch to sucrose than wheat and barley, in both of which sucrose strongly predominated (Fig. 2C). Loss of PTST2 roughly halved the starch content in barley and slightly reduced the sucrose content in wheat (*P*≤0.05), but effects on the starch:sucrose ratio were relatively minor in both species. In contrast, loss of PTST2 in *Brachypodium* caused a 10-fold reduction of starch content (*P*≤0.05) and no change in sucrose content. This led to a strong shift in the starch:sucrose ratio in favour of sucrose, and the total amount of starch and sucrose in *Bdptst2* was less than half of that in the wild type. Since most defects in granule initiation in Arabidopsis lead to ADP-glucose accumulation (Ragel *et al.*, 2013; Seung *et al.*, 2017), we extracted metabolites from leaves harvested at the end of the day and quantified ADP-glucose using LC-MS (Fig. 2D). Whereas loss of PTST2 had no effect on ADP-glucose levels in wheat or barley, it caused a 350-fold elevation of ADP-glucose in *Brachypodium* leaves.

**Fig. 3.**
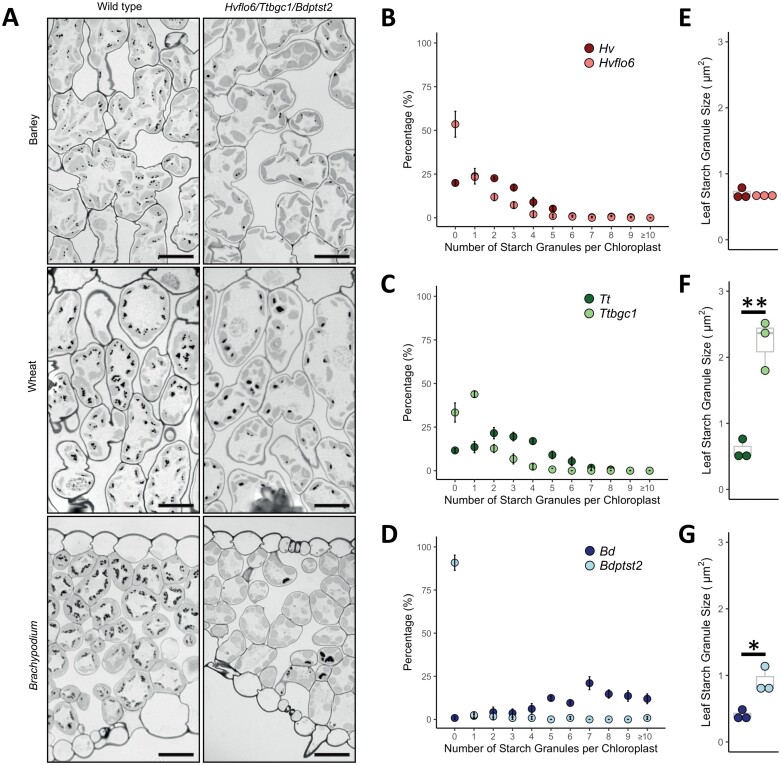
Leaf starch granule phenotypes of *ptst2* mutants. (A) Light microscope images of sections of leaves harvested at the end of the day of wild-type and *ptst2* plants of barley [*H. vulgare* (*Hv*); *Hvflo6*], wheat [*T. turgidum* (*Tt*); *Ttbgc1*], and *Brachypodium* [*B. distachyon* (*Bd*); *Bdptst2*]. Starch granules were stained dark with periodic acid–Schiff’s reagent, while chloroplasts were lightly stained with Toluidine Blue. Scale bars=20 μm. (B–D) The number of starch granules per chloroplast at the end of the day in wild-type and *ptst2* leaves (*n*=3) for (B) barley, (C) wheat, and (D) *Brachypodium*. The number of starch granule sections was quantified in light micrographs for at least 50 chloroplasts per biological replicate (*n*=3). Error bars are the SE of sections from three biological replicates. (E–G) Granule size at the end of the day in wild-type and *ptst2* leaves for (E) barley, (F) wheat, and (G) *Brachypodium*. At least 40 granules were quantified per biological replicate (*n*=3). An asterisk represents a significant change (*P≤0.05 and **P≤0.001) calculated using a Welch’s *t*-test.

To discover the impact of loss of PTST2 on starch granule initiation, we measured starch granule number per chloroplast and size (calculated as area) in leaf sections at the end of the day (Fig. 3). Loss of PTST2 markedly affected the number of starch granules per chloroplast in all three species. In barley, 80% of chloroplasts in wild-type leaves contained visible starch granules, whereas <50% contained starch granules in *Hvflo6* (Fig. 3B). In addition, the chloroplasts of *Hvflo6* where starch granules were visible had fewer granules than wild-type chloroplasts. In wheat, *Ttbgc1* had more than twice as many chloroplasts with no granule, or only one granule, compared with wild-type leaves (Fig. 3C). Again, when granules were visible, far fewer chloroplasts contained 2–6 granules in *Ttbgc1*. In *Brachypodium* almost all chloroplasts in wild-type leaves had visible granules, with most having 4–12 granules. In *Bdptst2*, only ~10% of chloroplasts contained visible starch granules, and these had variable numbers of granules per chloroplast (ranging from one to >10) (Fig. 3D). In addition to alterations in the frequency of granules per chloroplast, loss of PTST2 also resulted in changes in granule size in *Ttbgc1* and *Bdptst2*, but not in *Hvflo6* (Fig. 3E–G). Granules were on average four times larger (four times the cross-sectional area) in *Ttbgc1* and twice as large in *Bdptst2* as in wild-type plants (*P*≤0.05). In addition, a wide range of granule size within each *Bdptst2* biological replicate was noted.

In summary, *ptst2* mutations had diverse effects on leaf starch across the three grass species. In barley, the loss of PTST2 reduced the numbers of granules and starch content but did not affect granule size. In wheat, the loss of PTST2 reduced granule numbers but increased granule size, and there was no effect on starch content. In the *Brachypodium* mutant, although granule size was increased relative to the wild type, granule numbers and starch content were strongly reduced.

### Photosynthesis is impaired only in *Bdptst2*

**Fig. 4.**
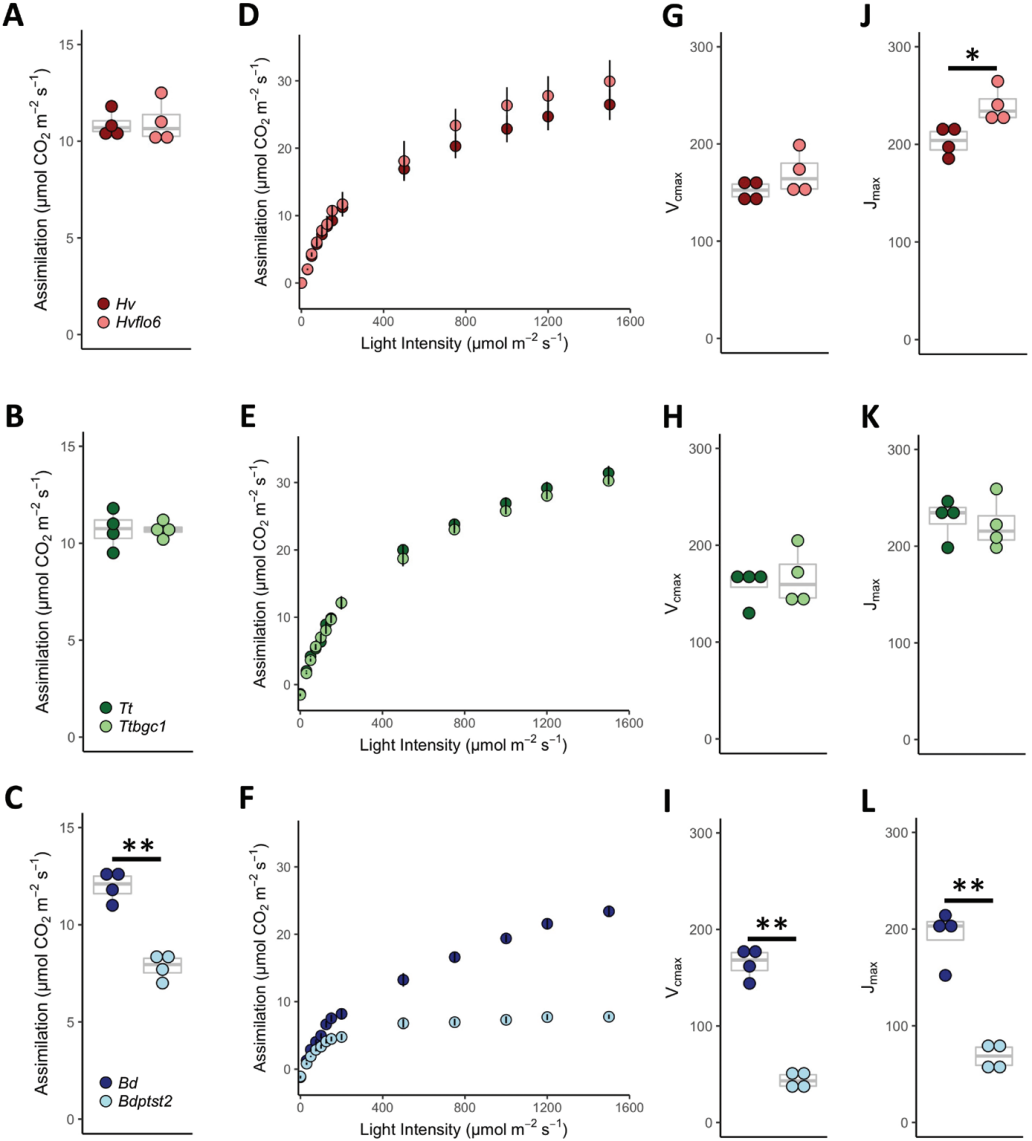
Physiological impact of *ptst2* mutations. CO_2_ assimilation under growth conditions for wild-type and *ptst2* leaves (*n*=4) for (A) barley, (B), wheat, and (C) *Brachypodium*. AQ curves from wild-type and *ptst2* leaves (*n*=4) for (D) barley, (E), wheat, and (F) *Brachypodium*. Error bars represent the SE. *V*_cmax_ (G, H, and I) and *J*_max_ (J, K, and L) calculated from *A*/*C*i curves (*n*=4) for barley, wheat, and *Brachypodium*, respectively. Barley [*H. vulgare* (*Hv*); *Hvflo6*], wheat [*T. turgidum* (*Tt*); *Ttbgc1*], and *Brachypodium* [*B. distachyon* (*Bd*); *Bdptst2*]. For dot plots, each dot represents a biological replicate, and the box and whisker plots illustrate the median, upper quartile, and lower quartile. An asterisk represents a significant change (*P≤0.05 and **P≤0.001) calculated under a Welch’s *t*-test.

To investigate how physiological functioning is impacted by the loss of PTST2, photosynthetic measurements were ­undertaken (Fig. 4). Only *Bdptst2* had a decreased assimilation rate when compared with the wild type under growth conditions (*P*≤0.05; Fig. 4A–C). Further, light response (AQ) curves showed that assimilation rates became light saturated substantially prior to the wild type only in *Bdptst2*, with light-saturated assimilation rates occurring at 500 µmol m^−2^ s^−1^ of light in the mutant (Fig. 4D–F). Assimilation against intracellular CO_2_ (*A*/*C*i) curves were used to calculate the maximum rate of carboxylation (*V*_cmax_) and maximum rate of electron transport (*J*_max_) (Fig. 4G–L). Both *V*_cmax_ and *J*_max_ were significantly reduced in *Bdptst2* relative to the wild type (*P*≤0.05; Fig. 4I, L). No significant differences were noted when comparing *Ttbgc1* with the wild type; however, there was a significant increase in *J*_max_ in *Hvflo6* when compared with the wild type (*P*≤0.05; Fig. 4J). In addition, total chlorophyll content was reduced only in *Bdptst2* (*P*≤0.05; Supplementary Fig. S2).

### Loss of PTST2 promotes granule initiations in the pericarp and endosperm of developing *Brachypodium* seeds

**Fig. 5.**
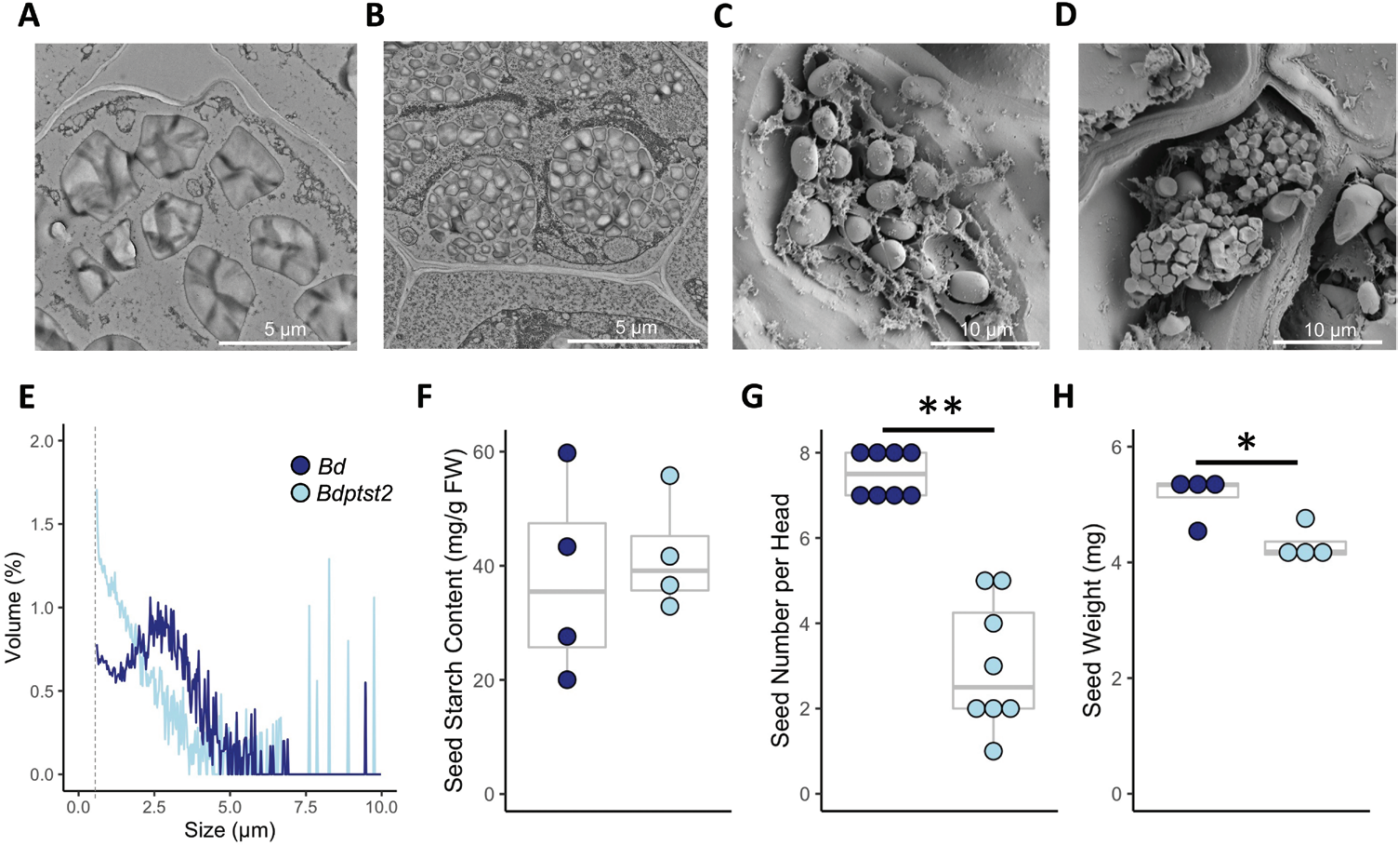
Compound granule formation in *Bdptst2* seeds. TEM images of the pericarp from seed sections at 3 DAF for (A) *Bd* and (B) *Bdptst2*. Scale bars=5 μm. SEM images of the endosperm from mature seed sections (C) *Bd* and (D) *Bdptst2*. Scale bars=10 μm. (E) Granule size distribution of starch extracted from mature seeds. The dotted line shows the lower limit of resolution for granule size (0.6 μm). (F) Starch content (*n*=4), (G) seed number per head (*n*=8), and (H) seed weight at maturity (*n*=4). Seed weight is an average of 15 seeds per biological replicate. *Bd* is wild type *B. distachyon*. For dot plots, each dot represents a biological replicate, and the box and whisker plots illustrate the median, upper quartile, and lower quartile. An asterisk represents a significant change (*P≤0.05 and **P≤0.001) calculated using a Welch’s *t*-test.

In the early stages of *Brachypodium* seed development, starch accumulates in the maternally derived pericarp. At later stages, starch declines in the pericarp and storage starch accumulates in the endosperm (Trafford *et al.*, 2013). Light microscopy and TEM at the earliest possible developmental stage [3 d after flowering (DAF)] showed the formation of compound-like structures in the *Bdptst2* pericarp, consisting of many small granules clustered in a circular shape (Fig. 5A, B; Supplementary Fig. S3). Granules in the pericarp of wild-type seeds were larger than those in mutant seeds, and they did not cluster into compound-like structures (Fig. 5A, B; Supplementary Fig. S3). Similarly, in mature seeds, starch granules in the endosperm were simple in the wild type but mostly compound in *Bdptst2* (Fig. 5C, D). Quantification of granule size distribution of starch purified from mature seeds showed that *Bdptst2* seeds had a higher proportion of small granules than wild-type seeds (Fig. 5E). However, total starch content of mature seeds was not significantly different between the mutant and wild type (Fig. 5F). It is also worth noting that the number of seeds per head was greatly decreased in the mutant compared with the wild type, and average seed weight was ~16% lower in the mutant (*P*≤0.05; Fig. 5G, H). In line with this decrease in weight, the mutant also produced significantly smaller seeds (*P*≤0.05), with the average seed length in the mutant (6.6 ± 0.1 mm) being ~12% shorter than in the wild type (7.5 ± 0.1 mm). Interestingly, despite the changes in seed size and starch morphology in the mutant, almost complete germination rates were observed in both genotypes.

### Transcriptomic differences between developing seeds of wild-type *Brachypodium* and *Bdptst2*

**Fig. 6.**
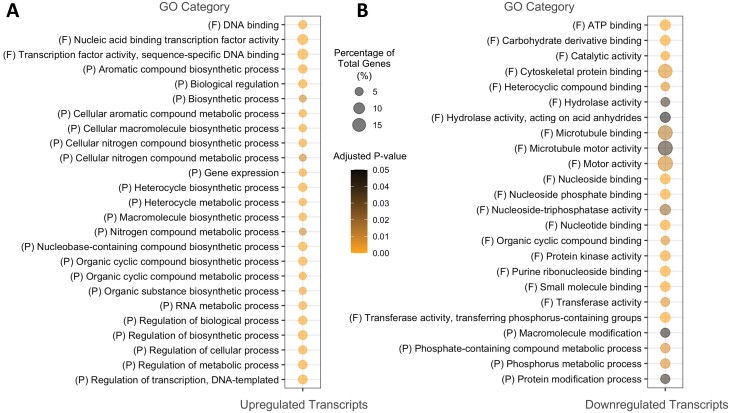
Changes in the transcriptome in seeds of *Bdptst2*. Significantly over-represented (adjusted *P*-value ≤0.05) Gene Ontology (GO) categories within the significantly (A) up-regulated and (B) down-regulated transcripts (FDR ≤0.01; *n*=4) identified in the seeds of *Bdptst2* (as compared with the wild type) at 6 d after flowering. The size of the circle represents the percentage of the total number of genes within that GO category, while the colour reflects the significance of each GO category. GO category aspects are either molecular function (F) or biological process (P).

To discover transcriptional differences between wild-type and mutant seeds that may be associated with simple versus compound granule formation, including potential changes in gene expression that can explain the greater number of initiations in the *ptst2* mutant, we performed RNA sequencing on developing seeds at 6 DAF. This stage was selected as it was the earliest at which the endosperm could be distinguished and contained starch granules (Supplementary Fig. S4). There were >1300 significantly differentially expressed genes (DEGs), with 588 transcripts up-regulated and 729 down-regulated in mutant relative to wild-type seeds (FDR ≤0.01; Supplementary Table S1). To gain information from this large number of DEGs, we identified over-represented GO (OR-GO) categories (adjusted *P*-value ≤0.05; Fig. 6A, B). Up-regulated OR-GO categories included many related to biosynthetic and metabolic processes and to transcription factor activity. The OR-GO categories within the down-regulated differentially expressed transcripts were more diverse; however, there were several related to protein modification and functioning. Carbohydrate derivative binding was also identified as a down-regulated OR-GO category.

**Fig. 7.**
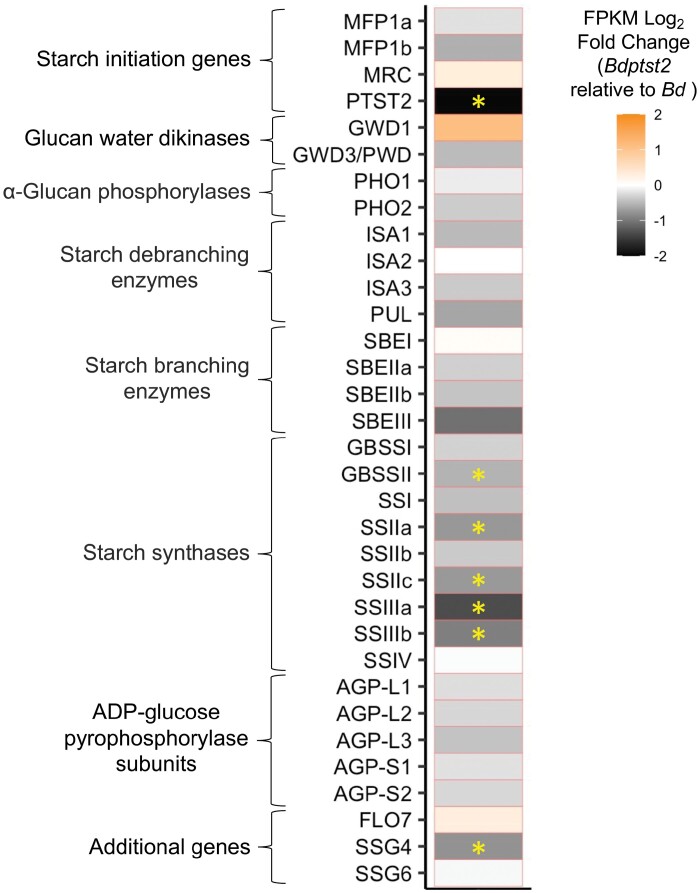
Transcript abundance of known starch genes in *Bdptst2*. FPKM log_2_ fold change (*Bdptst2* relative to *Bd*) of known starch genes in seeds at 6 d after flowering (*n*=4). *Bd* is wild-type *B. distachyon*. A yellow asterisk highlights transcripts that were identified as significantly differentially expressed (FDR≤0.05).

To investigate genes directly related to starch granule synthesis and regulation, transcript abundance of orthologues from known genes involved in these processes in Arabidopsis and rice were extracted (Fig. 7). The corresponding *B. distachyon* IDs are provided in Supplementary Table S2. As expected, transcript abundance of PTST2 was significantly and very strongly reduced (FDR ≤0.05; log_2_ FC of –1.97) in *Bdptst2* seeds. Five starch synthase genes were also significantly down-regulated (FDR ≤0.05) in *Bdptst2* seeds. In addition, SUBSTANDARD STARCH GRAIN4 (SSG4), which is known to affect compound granule morphology in rice (Matsushima *et al.*, 2014), had significantly decreased expression in *Bdptst2* seeds (FDR ≤0.05). While relatively strong log_2_ FCs were also observed for GWD and SBEIII, these were not significant at FDR ≤0.05.

## Discussion

### The loss of PTST2 causes distinct starch phenotypes in wheat and barley leaves

Although PTST2 is required for normal granule initiation in the endosperm of wheat and barley (Chia *et al.*, 2020; Hawkins *et al.*, 2021), its role in leaves has not yet been explored. In our study, *Hvflo6* and *Ttbgc1* mutants had distinct starch phenotypes in the leaves. Despite strong reductions in starch granule number per chloroplast, *Ttbgc1* maintained a similar starch content to the wild type, a phenotype that is likely to be linked to the production of larger granules (Figs 2, 3). PTST2-deficient mutants of Arabidopsis and rice also produce fewer, larger granules in leaves (Seung *et al.*, 2017; Zhang *et al.*, 2022)—although, unlike the barley, wheat, and Arabidopsis mutants, the rice mutant has a higher leaf starch content than wild-type rice. The barley mutant, *Hvflo6*, had reduced leaf starch content compared with the wild type, resulting from a high proportion of chloroplasts containing no starch granules, and no significant difference in granule size (Figs 2, 3).

Both *Hvflo6* and *Ttbgc1* showed a similar reduction in biomass when compared with the wild type (Fig. 1). The previously reported rice *flo6* and Arabidopsis *ptst2* mutants had no obvious growth phenotypes (Peng *et al.*, 2014; Seung *et al.*, 2017). Photosynthesis was not impaired in either the wheat or barley mutant; in fact, when compared with the wild type, *Hvflo6* had an increased maximum rate of electron transport (*J*_max_) (Fig. 4). Therefore, the reduction in starch content in *Hvflo6* is probably the cause of the reduction in its biomass. Although there was no change in starch content in *Ttbgc1*, there was a lower sucrose content in the mutant compared with the wild type (Fig. 2). The reason behind this reduction remains unknown. However, a reduction in sucrose content suggests lower sucrose availability for nocturnal mobilization, which could contribute to the reduced growth in the mutant.

### The loss of PTST2 is particularly deleterious for plant growth in Brachypodium

In contrast to wheat, barley, Arabidopsis, and rice (Peng *et al.*, 2014; Seung *et al.*, 2017; Chia *et al.*, 2020), loss of PTST2 in *Brachypodium* has severe, deleterious consequences. *Bdptst2* plants were pale, slow growing, and almost starchless, and they accumulated large amounts of ADP-glucose (Figs 1, 2). In Arabidopsis, ADP-glucose accumulation in the most severe granule initiation mutants (i.e. *ss4* and *ss3 ss4*) has deleterious consequences, including pale leaves and slow growth. These effects are attributed to limitation of adenylates available for photophosphorylation and hence reduced chloroplast function (Ragel *et al.*, 2013; Seung *et al.*, 2016). It is likely that a similar mechanism causes the pale phenotype and slow growth of *Bdptst2*, leading to the observed low rates of, and capacity for, CO_2_ assimilation. In contrast, *Hvflo6* and *Ttbgc1* leaves did not accumulate significantly more ADP-glucose than the wild type, and Arabidopsis *ptst2* plants were reported to have only modest increases in levels of ADP-glucose (Seung *et al.*, 2017). Thus, it appears that loss of PTST2 has a particularly strong effect on starch granule initiation and leaf physiology in *Brachypodium* compared with other examined species.

Two factors may contribute to the strong *ptst2* phenotype in *Brachypodium* compared with the other species. Firstly, there may be differences between species in the capacity to initiate granules in the absence of PTST2, resulting from differences in the complements of granule initiation proteins. In Arabidopsis, the ability of *ptst2* mutants to initiate at least one granule in most chloroplasts can be partially explained by the presence of a homologue, PTST3, since the *ptst2 ptst3* double mutant has more starchless chloroplasts than either single mutant and accumulates more ADP-glucose (Seung *et al.*, 2017). Arabidopsis STARCH SYNTHASE5 (SS5) also contributes a PTST2-independent mechanism of granule initiation, since *ptst2 ss5* mutants also have fewer granules than *ptst2* (Abt *et al.*, 2020). However, species in the Pooideae have no orthologues of PTST3 or SS5 so these components cannot be involved in granule initiation in wheat, barley, or *Brachypodium*. The relatively mild phenotypes in wheat and barley mutants indicate that other, unknown components can participate in granule initiation in the absence of PTST2: it may be the case that the much stronger phenotype in *Brachypodium* stems from interspecific differences in expression of these unknown components. This aspect is discussed further below.

Secondly, *Brachypodium* may be more sensitive to defects in granule initiation than barley and wheat due to a greater reliance on leaf starch as a carbohydrate reserve for night-time maintenance. For example, in a 12 h day, 12 h night cycle, *Brachypodium* leaves turn over ~20% more starch than sucrose, whereas barley leaves turn over three times more sucrose than starch (Müller *et al.*, 2018, Preprint). There are numerous additional reports of very high sucrose to starch turnover ratios in wheat and barley leaves (Gordon *et al.*, 1980; Shaikh *et al.*, 2000). Additional carbohydrate reserves could also be present in wheat and barley, and not *Brachypodium*, such as fructans (Wagner *et al.*, 1986; Farrar, 1989). It is also possible that the severe effects of ADP-glucose accumulation on chloroplast function have further knock-on effects on granule initiation.

The extent to which the loss of PTST2 negatively affects vegetative growth and physiology has important consequences on targeting granule initiation to modify the morphology of seed starch to improve its industrial applications (Chen *et al.*, 2021). Our results are promising for the application of granule initiation mutants in wheat and barley, and potentially in other cereals that primarily turn over sucrose in leaves. Although *ptst2* mutants in wheat and barley had slightly reduced biomass, the phenotype was much less severe than that of the *Brachypodium* mutant, and the beneficial impacts on seed starch morphology may outweigh the limited adverse effects on growth. Our next steps are to determine the field performance of these wheat and barley mutants.

### Tissue-specific effects of the PTST2 mutation in *Brachypodium*

Mature *Brachypodium* seeds have much lower starch contents than the seeds of wheat, barley, and many other grasses in the Pooideae. Starch contents from ~4% to 15% of seed weight have been reported (Opanowicz *et al.*, 2011; Tanackovic *et al.*, 2014), whereas the starch content of mature wheat and barley seeds is typically 60–70% of the seed weight (Hannah and James, 2008). *Brachypodium* endosperm, and the pericarp at early stages of seed development, contains simple granules, probably arising from a single initiation event per amyloplast, whereas the majority of species in the Pooideae produce either compound granules or, in the Triticeae, two distinct types of granule of different average sizes—a bimodal size distribution (Matsushima *et al.*, 2013; Tetlow and Emes, 2017). Whereas we and others (Chen *et al.*, 2014) found a unimodal distribution of granule sizes in mature *Brachypodium* seeds (Fig. 5), there are also reports of bimodal size distributions (Matsushima *et al.*, 2013; Tanackovic *et al.*, 2014). It is clear, however, that *Brachypodium* does not possess the A- and B-type granules found in the Triticeae, where the two types differ not only in size, but also in shape, location, and time of formation (Seung and Smith, 2019).

*Bdptst2* plants were able to set seeds with normal starch content when expressed on a per weight basis (Fig. 5). However, seed weight was decreased in the mutant, probably due to the reduction in seed size. It is possible that the synthesis of starch and other major storage reserves, such as β-glucans (Trafford *et al.*, 2013), is affected by the reduced source capacity in the mutant that results from reduced assimilation rate and slow growth, such that overall starch content remains similar. It should also be noted that the total sink strength of seeds was much lower in the mutant than in the wild type, as the number of seeds per plant was severely reduced. Since aborted or shrunken seeds were not observed, the reduction in seed number may be due to either defects in pollen development or the abortion of ear primordia prior to fertilization. It has previously been shown that defects in pollen starch can occur in starch initiation mutants (Hawkins *et al.*, 2021; Zhang *et al.*, 2022). There is also the possibility that primordial abortion prior to fertilization is occurring due to reduced carbon assimilation, and hence assimilate export, a trait previously reported in cereals (Boyer and Westgate, 2004; Ferguson *et al.*, 2021).

In both the pericarp and endosperm, *Bdptst2* produced distinctive compound starch granules arising from supernumerary granule initiations per amyloplast (Fig. 5). This phenotype is like that previously reported in the endosperm of barley and wheat *ptst2* mutants, which have ‘semi-compound’ or compound granules in place of most A-type granules (Chia *et al.*, 2020; Hawkins *et al.*, 2021). In rice, a species that normally produces compound granules, the loss of PTST2 leads to heterogenous amyloplasts with highly variable numbers of initiations within each compound granule (Peng *et al.*, 2014). Thus, PTST2 appears to play a major role in establishing all three major patterns of granule initiation (simple, compound, and bimodal) in grass endosperms.

We have demonstrated that loss of PTST2 can have distinct effects on granule initiation in different organs and species. In all three species examined, loss of PTST2 led to overall reductions in the number of granules initiated in leaves, and increased numbers of granule initiations in the endosperm. It is interesting to note that while most chloroplasts in the *Brachypodium* mutant contained no starch granule, those that did contained varying numbers of granules, sometimes even exceeding 10 granules (Fig. 3). Similarly, normal simple starch granules that appeared to arise from single initiations were occasionally seen in both the pericarp and endosperm of the mutant (Supplementary Fig. S3). The common observation between all organs and species is that PTST2 is required to establish correct numbers of granules.

### How do starch granules initiate in the absence of PTST2?

A remaining question from our study is how granules initiate in the absence of PTST2 in the endosperm. In Arabidopsis, rice, and wheat, PTST2 is proposed to act in granule initiation with SS4 (Seung *et al.*, 2017; Hawkins *et al.*, 2021; Zhang *et al.*, 2022). The lack of PTST2 may reduce SS4 function, meaning other enzymes that can elongate oligosaccharides, such as other starch synthases (SSs) and plastidial phosphorylase (PHS1), may initiate granules (Hwang *et al.*, 2010; Brust *et al.*, 2013; Cuesta-Seijo *et al.*, 2016). It appears that the efficiency of these alternative initiation mechanisms is greater in seeds than in leaves since there was no significant decrease in total starch content of *Bdptst2* seeds. Mutation of *ptst2* in *Brachypodium* resulted in large transcriptional changes in developing seeds (Figs 6, 7). Despite the functional connection between SS4 and PTST2 in Arabidopsis, SS4 expression was not altered in the *Bdptst2* mutant, and we did not see significantly higher expression of any SS or PHS1 in the mutant seeds that could explain the increased number of initiations (Fig. 7). In fact, there was a significant reduction in the expression of several SS genes in *Bdptst2*. However, as detectable expression was still noted in *Bdptst2* seeds for all SS genes, it remains possible that they contribute to granule initiation in the mutant, and that the activity of such enzymes may not be regulated at the transcriptional level. Only one additional gene known to have a function related to starch synthesis had significantly different expression in the mutant, SUBSTANDARD STARCH GRAIN4 (SSG4). SSG4 encodes the rice orthologue of Arabidopsis TIC236 that links the protein import translocons on the inner and outer plastid membranes. Mutants deficient in SSG4 have highly abnormal compound granules (Matsushima *et al.*, 2014), and it is possible that its decreased expression contributes to compound granule formation in the mutant—thus representing an attractive gene for further characterization in *Brachypodium*.

## Supplementary data

The following supplementary data are available at *JXB* online.

Fig. S1. Confirmation of the *Brachypodium distachyon ptst2* mutant.

Fig. S2. Chlorophyll content of leaves of wild-type and *ptst2* plants.

Fig. S3. Light microscope images of pericarp development for *Bd* and *Bdptst2*.

Fig. S4. Light microscope images of grain development for *Bd* and *Bdptst2.*

Table S1. Transcript abundance changes between *Bd* and *Bdptst2* seeds at 6 DAF.

Table S2. *Brachypodium distachyon* IDs for known starch genes of interest.

erac268_suppl_Supplementary_Figures

erac268_suppl_Supplementary_Tables

## Data Availability

Raw RNA sequencing reads and genome sequencing reads are available within GenBank under the BioProject ascension PRJNA798879.
