# Supplementary material for: Loss of PROTEIN TARGETING TO STARCH 2 has variable effects on starch synthesis across organs and species
Source: J Exp Bot. 2022 Jun 18;73(18):6367–79. doi: 10.1093/jxb/erac268 (PMC9578351; doi:10.1093/jxb/erac268)
Supplement: erac268_suppl_Supplementary_Figures [file erac268_suppl_supplementary_figures.pdf]

A

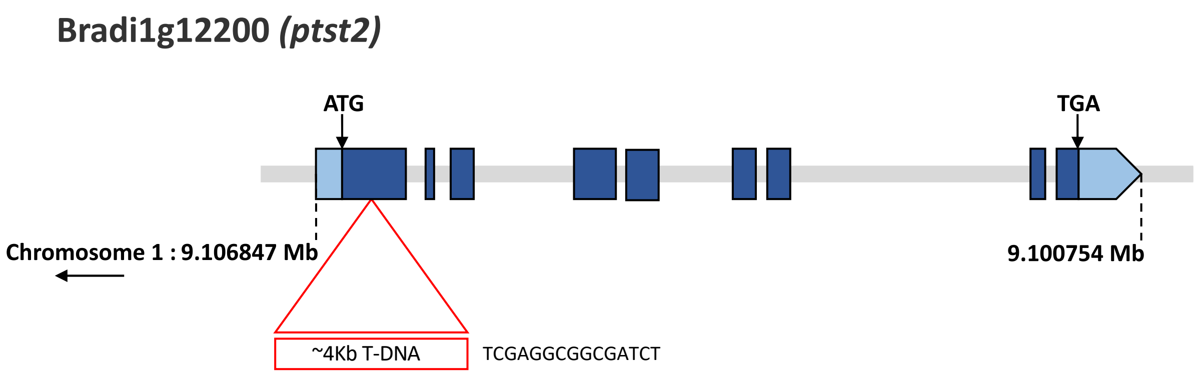

B

>T-DNA Insert Sanger Sequencing
GATACTCGTAAACTACATATCGAGCCGGAGCATAAAGTGTAAAGCCTGGGGTGCCTAATGAGTGAGCTAACT
CACATTAATTTGTCTAAGCGTCAATTTGTTTACACCACAATATATCGAGGCGGCGATCTACGACTTCATGCGCA
GCTCCGACAAGCCCGGCGCGTTCCCCACCCGCGCGGAGCTCCTCGCCGCGGGCCGCGCCGACCTCGCCCGGG
CGGTCGAGTCCAGCGGAGGGTGGCTCTCCCTCGGCTGGTCCTCGGGAGACTCCGTCTCGCCGTGCGTTACGG
CGTCGGCCTCGTCATTGGACGGCGGCGCCGGCGTGCACCCTGACTACCCTCCCGAGGCGGCGGGCACCTCGG
ACCTAGCACCGGGCGCGGAGGGGGCTTCCTGGAGGTGTGTAATTTGAGTCGCTCACTGTGCTGCCCTCGTCT
GTTCCGCCCTTCTCCGTGTAACCTCTTTCTGAATTCGGCGTGTTGTAGGGAGCCAGAAGCGTCGCCGTCTGGG
AGGCCGCCGGAGACGGAGGGGACAGAGTGCGTGCCTTCTAAATCTCTTTCTCTATTTTTTTTTTCTTCTCTTGG
GTTGGTTCCCTTATTGTGTCGTTGCTTTTTGCACGGTGATTGATTGCAGGGAGGTGGTTTCTGGAGCAGGCCT
GGAGGGGATGCTCACCAGGCTTCAGAGAGAGAGGGAGCGTGCGCGGCCACCACCACGGAGCAATAACCGA
GGG

C

BdPTST2_Forward	TCCAGAGACTAGCGTAGCGT
BdPTST2_Reverse	GAGAAGCGAGACAGCACGTA
Bd_T3_T-DNA_Insert_Forward	AGCTGTTTCCTGTGTGAAATTG

D

Insert location					
Chromosome	Position	Strand	Insert	Support reads (pair+softclip)	Confidence
1	9106356	+	T3 T-DNA Insert	13+12	high

Supplementary Figure 1 – Gene model for (**A**) Bradi1g12200 (PTST2). (**B**) Sequence of T-DNA flanking region determined by Sanger sequencing. Nucleotides corresponding to the T-DNA are shown in red, while those that correspond to PTST2 are shown in blue (exon) and black (intron). (**C**) Primers used to genotype *Bdptst2* mutants. (**D**) Location and number of reads mapped to the single T-DNA insert present in *ptst2* mutant.

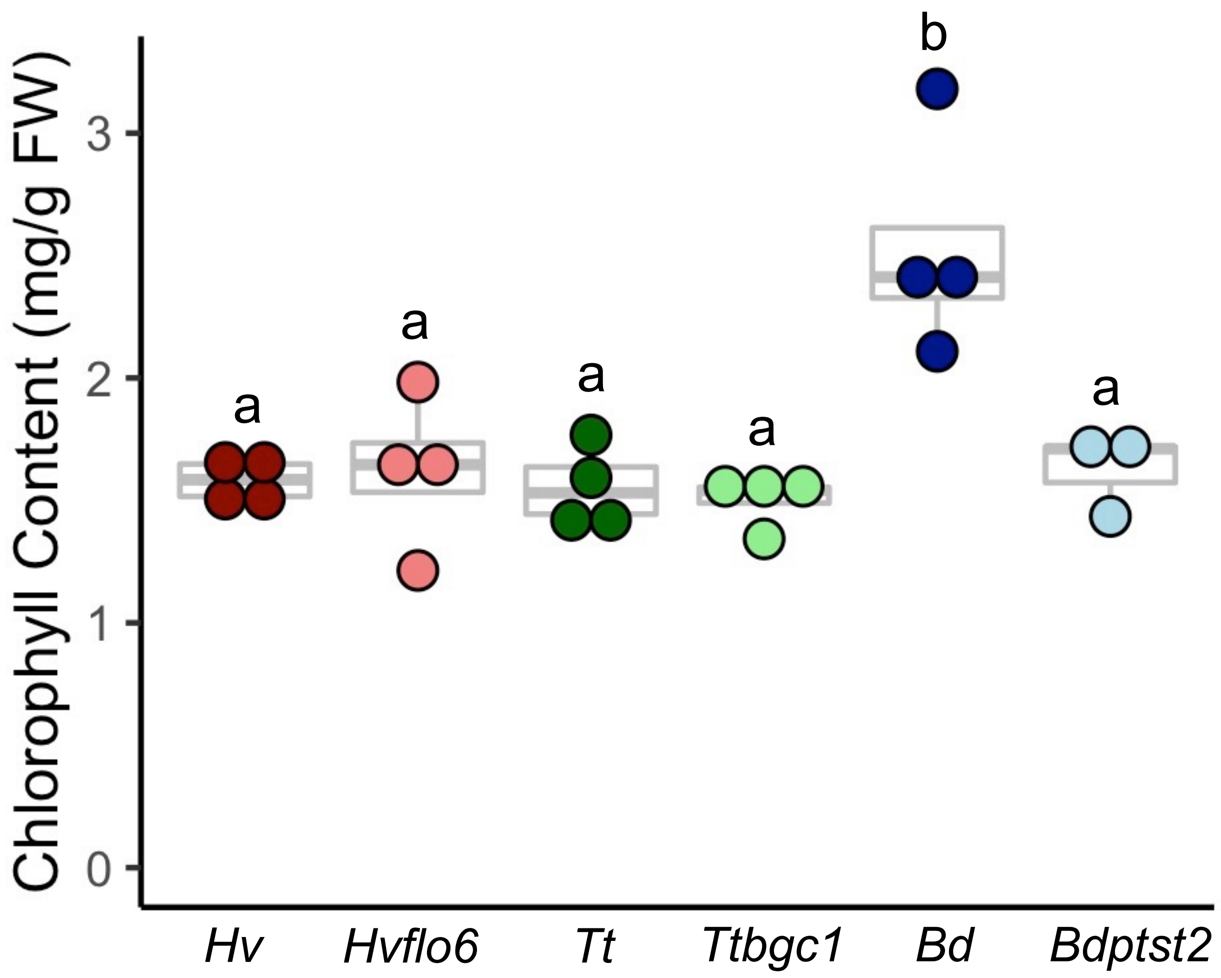

Supplementary Figure 2 – Chlorophyll content of leaves of wild type and *ptst2* plants (n=4) of barley (*H. vulgare* (*Hv*); *Hvflo6*), wheat (*T. turgidum* (*Tt*); *Ttbgc1*) and *Brachypodium* (*B. distachyon* (*Bd*); *Bdptst2*). Box and whisker plots show the median, upper quartile and lower quartile. Significance is indicated where two letters differ ($P \leq 0.05$) calculated using a two-way ANOVA and a Tukey post hoc test.

A **B**

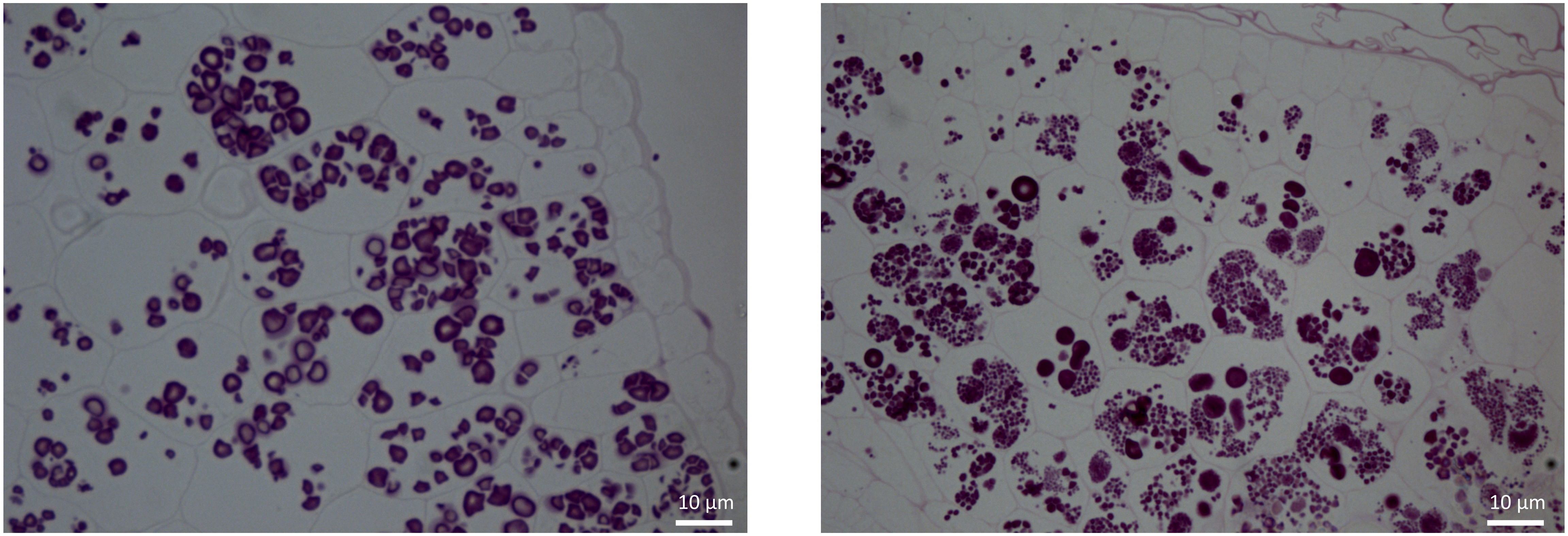

Supplementary Figure 3 – Light microscope images of the pericarp at 3 days after flowering from (**A**) wild type *B. distachyon* and (**B**) *Bdptst2*. Sections were stained with periodic acid-Schiff's reagent. Bars = 10 µm.

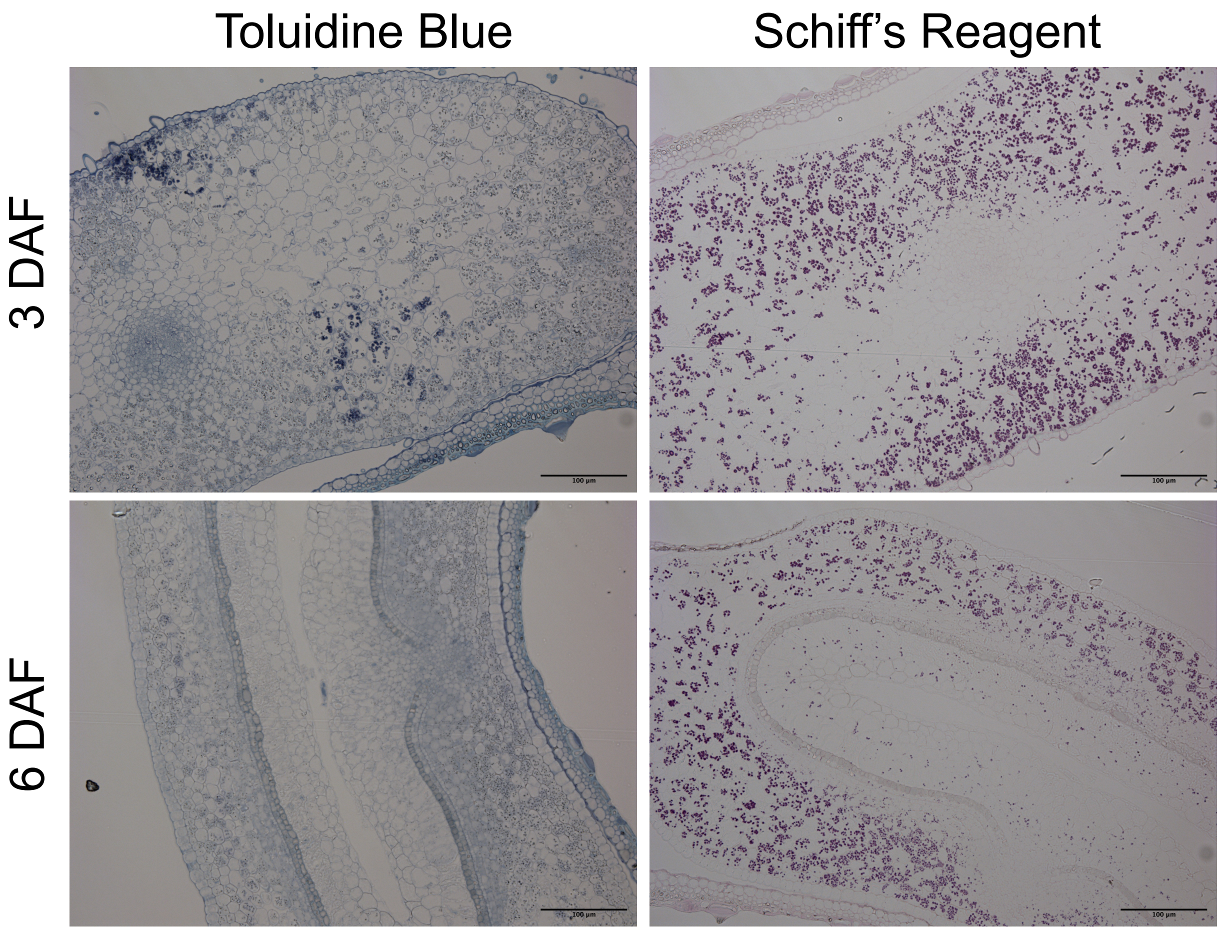

Supplementary Figure 4 - Light microscope images of wild type *B. distachyon* seed sections at 3 days after flowering (DAF) and 6 DAF stained with either toluidine blue or periodic acid-Schiff's reagent. Bars = 100 µm.
